# Rapid and Specific Detection of Active SARS-CoV-2 With CRISPR/Cas12a

**DOI:** 10.3389/fmicb.2021.820698

**Published:** 2022-01-28

**Authors:** Xinyi Liu, Yanhua Li, Xin Wang, Yifan Song, Lina Wu, Benyuan Yu, Xiaodong Ma, Peixiang Ma, Ming Liu, Xingxu Huang, Xinjie Wang

**Affiliations:** ^1^Shenzhen Branch, Guangdong Laboratory of Lingnan Modern Agriculture, Genome Analysis Laboratory of the Ministry of Agriculture and Rural Affairs, Agricultural Genomics Institute at Shenzhen, Chinese Academy of Agricultural Sciences, Shenzhen, China; ^2^Guangzhou Laboratory, Bio-Island, Guangzhou, China; ^3^Jiangsu Co-innovation Center for Prevention and Control of Important Animal Infectious Diseases and Zoonosis, College of Veterinary Medicine, Yangzhou University, Yangzhou, China; ^4^School of Life Sciences and Technology, ShanghaiTech University, Shanghai, China; ^5^School of Life Sciences, Fudan University, Shanghai, China; ^6^School of Food Science and Pharmaceutical Engineering, Nanjing Normal University, Nanjing, China; ^7^Animal, Plant and Food Inspection Center of Nanjing Customs District, Nanjing, China; ^8^Institute for Brain Research and Rehabilitation, Guangdong Key Laboratory of Mental Health and Cognitive Science, Center for Studies of Psychological Application, South China Normal University, Guangzhou, China; ^9^Shanghai Key Laboratory of Orthopedic Implants, Department of Orthopedic Surgery, Shanghai Ninth People’s Hospital, Shanghai Jiao Tong University School of Medicine, Shanghai, China; ^10^State Key Laboratory of Respiratory Disease/National Clinical Research Center for Respiratory Disease/National Center for Respiratory Medicine/Guangzhou Institute of Respiratory Health/The First Affiliated Hospital of Guangzhou Medical University, Guangzhou, China

**Keywords:** CRISPR-based detection, CRISPR/Cas12a, virus subgenome, COVID-19, SARS-CoV-2

## Abstract

Rapid and sensitive nucleic acid detection of SARS-CoV-2 has contributed to the clinical diagnosis and control of COVID-19. Although detection of virus genomic RNA (gRNA) has been commonly used in clinical diagnosis, SARS-CoV-2 gRNA detection could not discriminate between active infectious virus with remnant viral RNA. In contrast to genomic RNA, subgenomic RNAs (sgRNAs) are only produced when the virus is actively replicating and transcription, detection of sgRNA could be an indication to evaluate infectivity. CRISPR/Cas-based nucleic acid detection methods have been considered potential diagnostic tools due to their intrinsic sensitivity, specificity and simplicity. In this study, to specifically detect active virus replication, we developed a CRISPR-based active SARS-CoV-2 (CRISPR-actCoV) detection strategy by detecting sgRNAs of SARS-CoV-2. CRISPR-actCoV with CRISPR Cas12a-assisted fluorescence reporter system enables detection of sgRNAs at 10 copies in 35 min with high specificity and can be read out with naked eyes. Further, we performed CRISPR-actCoV mediated sgRNA detection in 30 SARS-CoV-2 potentially infected clinical samples, and 21 samples were SARS-CoV-2 sgRNA positive. A quantitative RT-PCR assay was also performed to detect gRNA of SARS-CoV-2 in parallel. Among the 30 clinical samples, 27 samples were gRNA positive. Taken together, CRISPR-actCoV provides an alternative for rapid and accurate detection of active SARS-CoV-2 and has great significance in better response of coronavirus causing epidemic disease.

## Introduction

Severe acute respiratory syndrome coronavirus-2 (SARS-CoV-2), the cause of COVID-19 respiratory disease, has spread worldwide and affected global health and the world economy. Rapid and accurate nucleic acid detection of SARS-CoV-2 has contributed to the early diagnosis and treatment of COVID-19 (Chu et al., 2020; Orive et al., 2020; Seo et al., 2020; Wang et al., 2020c). The commonly reported nucleic acid detection methods, including quantitative real-time PCR (qRT-PCR), isothermal amplification-based detection and CRISPR-based detection, mainly target viral gRNA of SARS-CoV-2 genes, such as E gene, N gene, or orf1a/b gene (Broughton et al., 2020; Dao Thi et al., 2020; Wang et al., 2020c,f). The presence or absence of gRNA has been used to indicate virus contamination or infection (Chia et al., 2020; Yu et al., 2020). However, viral gRNA might still be detectable in months after clinical recovery (Xiao et al., 2020), and detection of gRNA does not prove the presence of active viral replication or infectious virus. It can not distinguish replicable virus from remnant viral RNA or laboratory-generated non-hazardous nucleic acids (Robinson-McCarthy et al., 2021).

SARS-CoV-2 is an enveloped, positive-sense, single-stranded RNA beta-coronavirus and belongs to the *Coronaviridae* family (Gordon et al., 2020). Coronavirus RNA-dependent RNA synthesis needs the transcription of a collection of sgRNAs that encode the viral structure proteins, and the presence of sgRNAs provides evidence of replicative and intact viruses (Sola et al., 2015). Since sgRNAs of SARS-CoV-2 are only produced when the virus is actively replicating and transcription (Sztuba-Solińska et al., 2011; Kim et al., 2020), detection of sgRNAs can distinguish replicable and intact viruses from remnant viral RNAs, thus providing essential information for better containment of COVID-19. Detection of SARS-CoV-2 sgRNAs has been performed in hospitalized patients of COVID-19 using qRT-PCR (Moreira et al., 2020; Wolfel et al., 2020; Rodríguez-Grande et al., 2021). qRT-PCR-based nucleic acid detection is most commonly used and is considered a gold standard for diagnosis. However, the requirement of dedicated instruments and well-trained technicians limit its on-site diagnostic application, especially in the resource-limiting region (Yin et al., 2021). Therefore, nucleic acid-based diagnostic strategies combining sensitivity, specificity, and flexibility are expected for point-of-care testing (POCT).

The clustered regularly interspaced short palindromic repeats (CRISPR) and CRISPR associated proteins (Cas) systems have been considered to have great potential in diagnostic, thus revolutionizing the nucleic acid detection area (Wang et al., 2020b; Lv et al., 2021). The most commonly used Cas proteins in the CRISPR/Cas-based nucleic acid detection system are Cas9, Cas12, Cas13 and Cas14 (Wang et al., 2020e). Among these, Cas12a mediate a robust, non-specific single-strand DNA (ssDNA) cleavage upon specific target recognition (Chen et al., 2018; Li et al., 2018a). Nucleic acid detection systems based on CRISPR/Cas12a have been established to detect various targets, including bacteria, viruses, cancer mutations, and others (Li et al., 2018b,^2019^; Wang et al., 2020d; Xiong et al., 2020; Lee Yu et al., 2021; Ma et al., 2021). Herein, we reported a CRISPR/Cas12a based nucleic acid detection strategy named CRISPR-actCoV (CRISPR-based active SARS-CoV-2 detection), which detects active SARS-CoV-2 sensitively and precisely. This study could provide useful information for the containment of epidemics caused by viruses, like SARS-CoV-2.

## Materials and Methods

### Clinical Samples and Ethics Statement

The study was approved by the Scientific Research Ethics Review Committee of the First Affiliated Hospital of Guangzhou Medical University. All the clinical samples used in this study were collected and treated in strict accordance with the standard operation for COVID-19 by the WHO and Chinese CDC. Informed consent was obtained from all the patients involved. Nasopharyngeal swab samples were collected from all patients at admission. The clinical sample RNA was extracted using a QIAamp RNA Viral Kit (Qiagen, 52904) in a biosafety level II laboratory. In brief, the nasopharyngeal swab was dissolved in an extraction buffer, and the viral RNA extraction was performed. After the wash step, the RNA was eluted in 60 μl RNase-free H_2_O.

### Nucleic Acid Preparation

For SARS-CoV-2 subgenome validation, nucleic acid fragments containing subgenomic sequences of *Orf3a, E, M, Orf6, Orf7a, Orf8* and *N* genes were amplified from cDNA of a diagnosed patient using reverse transcription PCR (RT-PCR, E045-01A, Novoprotein, China) and sequenced. The primers used to amplify SARS-CoV-2 (GenBank: MN908947) subgenomic sequences were designed and synthesized by GenScript (Nanjing, China). The primer sequences were listed in Supplementary Table 1.

The specific crRNAs targeting *E, Orf7a* and *N* genes of SARS-CoV-2 were synthesized by GenScript, and the crRNA sequences were listed in Supplementary Table 2. The nucleic acid fragments containing *E, Orf7a* and *N* subgenomic sequence of SARS-CoV-2 were synthesized by GenScript and then cloned into the pUC57 vector with a T7 primer. The sgRNAs of SARS-CoV-2 *E, Orf7a and N* were transcribed using the MEGAshortscript T7 Transcription Kit (Thermo Fisher, AM1354) and purified with the MEGAclear Kit (Thermo Fisher, AM1908) according to the manufacturer’s instructions. Transcribed RNAs were aliquot and stored at −80°C till used. The primers used for *in vitro* transcription are listed in Supplementary Table 3.

Total cellular RNA was extracted from HCT116 and A549 cells using a total RNA extraction reagent (Vazyme, R401-01), following the manufacturer’s instructions. Saliva RNA was extracted from the saliva sample using QIAzol Lysis Reagent (QIAGEN, 79306) following the manufacturer’s instructions. All RNAs were stored in aliquots at −80°C till used.

### Reverse Transcription Isothermal Amplification

The RT-RPA assays of the *E, Orf7*a and *N* sgRNAs were performed with a commercial RT-ERA kit (GenDx Biotech Co., Ltd.) according to the manufacturer’s instructions. The RT-RPA primers (Supplementary Table 4) were designed, and the RT-RPA reaction was performed as previously described (Wang et al., 2020f). In brief, a 50 μL reaction consisting of 2 μL RT-RPA-F (forward primer, 10 μM), 2 μL RT-RPA-R (reverse primer, 10 μM), 2 μL RNA template, and 2.5 μL magnesium acetate (280 mM) was incubated at 39°C for 20 min. Then, the RT- RPA reaction was transferred to the CRISPR/Cas12a-mediated nucleic acid detection assay.

### Cas12a-Mediated Nucleic Acid Detection

The CRISPR/Cas12a-mediated nucleic acid detection assays were performed according to the previous reports (Wang et al., 2020f). The Lachnospiraceae bacterium Cas12a (LbCas12a) was expressed and purified. A single-strand DNA reporter labeled with FAM and BHQ1 was synthesized by Genscript. 200 ng LbCas12a protein with 2 μL of RT-RPA product, 25 pM ssDNA FQ DNA reporter and 1 μM crRNA were subjected to detection assays in a 20 μL reaction system. The fluorescence was detected under 485 nm light and photographed with a mobile phone camera as reported (Wang et al., 2020f).

### Quantitative Real-Time PCR and DNA Sequencing of Clinical Samples

RNA was extracted from the clinical samples, and cDNA was synthesized using the kit from Thermo Fisher (Catalog Number: 18091050) according to the manufacturer’s manual. Quantitative real-time PCR of clinical samples was performed using HiScript II U + One Step qRT-PCR Probe Kit (Vazyme Biotech Co., Ltd.) according to the manufacture’s manual. The probe and primers for quantitative real-time PCR were synthesized by Genscript, and sequences were listed in Supplementary Table 5. Further, an NMPA-granted commercial kit (Liferiver, China) was used for these clinical samples’ nucleic acid detection assay. The assay was performed according to the manufacturer’s manual. In brief, extracted RNA was subjected to a two-step quantitative real-time PCR, the fluorescent signal of FAM and TexasRed was collected and Ct value < 43 was treated as positive.

### Statistical Analysis

All experimental results were shown as Mean ± SD unless stated otherwise. Statistical significance for comparisons of more than two groups was determined using one-way ANOVA. Significance was considered as *p < 0.05; ^**^p < 0.01. Statistical analyses were carried out with GraphPad Prism 7.0.

## Results

### Amplification and Verification of SARS-CoV-2 Subgenome in a Diagnosed Clinical Sample

**FIGURE 1:**
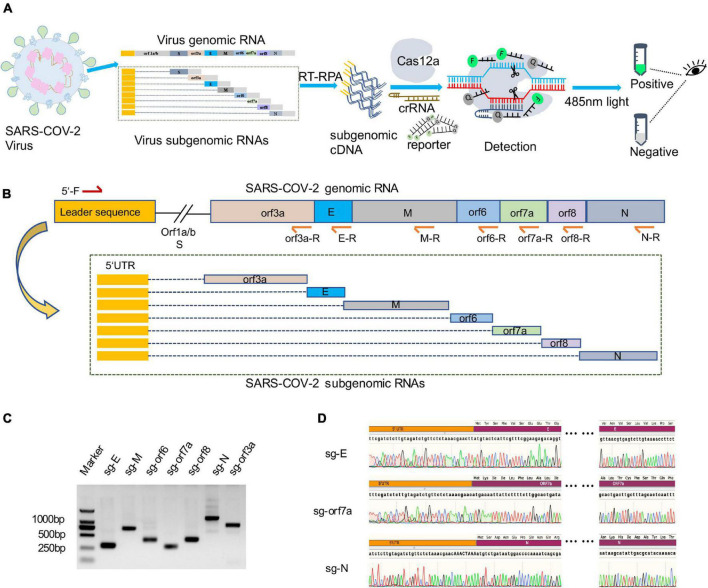
Amplification and verification of SARS-CoV-2 subgenome in a diagnosed clinical sample. **(A)** Schematic illustration of CRISPR/Cas12a based detection of active SARS-CoV-2 (CRISPR-actCoV). Subgenomic RNA was specifically amplified by RT-RPA and then subjected to CRISPR/Cas12a mediated fluorescence reporter assay, and the results can be read by naked eyes under 485nm light. **(B)** Schematic diagram of SARS-CoV-2 subgenomic RNAs (sgRNAs) and the location of primers used for sgRNAs amplification. The forward primer (5′-F) was designed to target a common 5′ leader sequence, the reverse primers (orf3a-R, E-R, M-R, orf6-R, orf7a-R, orf8-R, N-R) were designed to target each gene body. **(C)** Image of DNA agarose gel electrophoresis of amplified nucleic acid fragments containing SARS-CoV-2 sgRNA sequence. Marker, DL2000 DNA marker. sg-E, sg-M, sg-orf6, sg-orf7a, sg-orf8, sg-N, sg-orf3a, the nucleic acid fragments containing sgRNA of *E* gene, *M* gene, *orf6* gene, *orf7a* gene, *orf8* gene, *orf3a* gene. **(D)** Representative chromatograph of Sanger sequencing of amplified sgRNAs.

sgRNAs of SARS-CoV-2 are only produced during viral replication and are poorly packaged into mature virions, thus they have been proposed to represent a potential marker for active infection and viral replication (Immergluck et al., 2021). To specifically and rapidly detect viral replication, we developed a CRISPR-actCoV detection strategy by combining RT-RPA with CRISPR-Cas12a assisted fluorescence reporter system (Figure 1A). Virus sgRNAs were specifically amplified by RT-RPA to obtain enough DNA substrates using specific primers, and the amplicons were then subjected to CRISPR-Cas12a detection assays. In the reaction, purified Cas12a protein, specific crRNA, amplified DNA, and a single-strand DNA reporter labeled with FAM and BHQ1 were included. Upon target recognition and cleavage, the non-specific ssDNA cleavage activity of Cas12a was triggered, and fluorophores were released from the quencher. Then the detection results can be read by naked eyes under 485 nm light (Figure 1A).

Each sgRNA of coronavirus contains the common 5′-leader sequence fused to the gene body (Sola et al., 2015; Brant et al., 2021). To verify the existence of SARS-CoV-2 sgRNAs in a clinically diagnosed sample, we performed specific amplification of sgRNAs of *orf3a*, *E*, *M*, *orf6*, *orf7a*, *orf8*, and *N* gene. The specificity of amplification was ensured by primer designing, and the forward primer was designed to target the 5′ leader sequence and reverse primers were designed to target each gene body (Figure 1B). The agarose gel electrophoresis results demonstrated the existence of SARS-CoV-2 sgRNAs (Figure 1C). This result was further confirmed by Sanger sequencing of amplicons (Figure 1D and Supplementary Figure 1).

### Design and Evaluation of crRNAs Targeting SARS-CoV-2 Subgenomic RNAs

**FIGURE 2:**
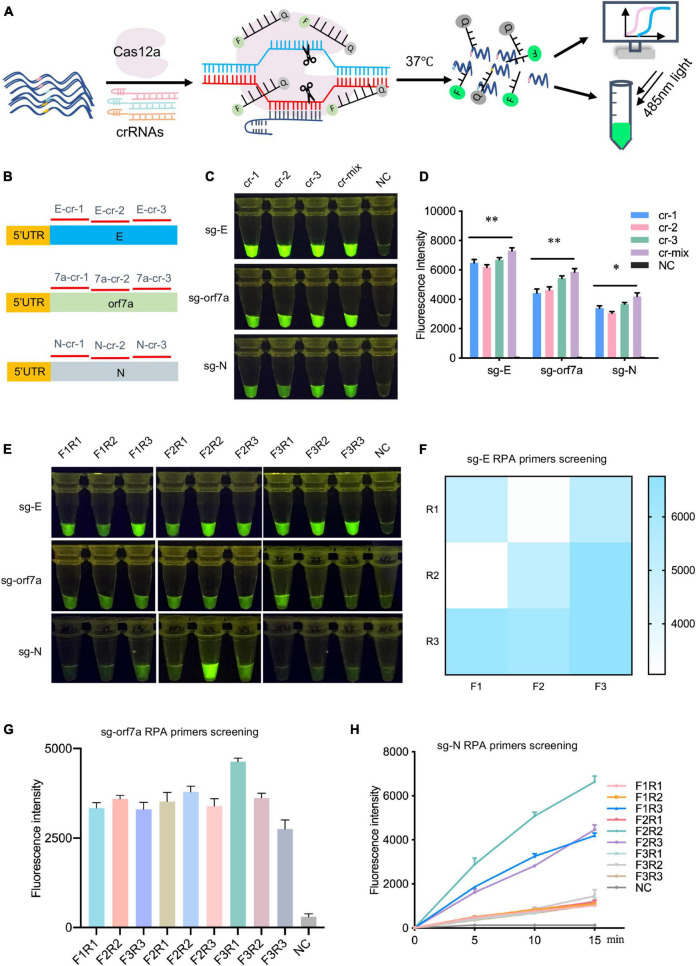
Screening of crRNAs and RT-RPA primers targeting sgRNA of SARS-CoV-2 *E*, *orf7a* or *N*. **(A)** Schematic illustration of crRNAs design and screening. For each target, three crRNAs were designed and synthesized, the efficiency of crRNAs was evaluated using CRISPR/Cas12a mediated fluorescence reporter assay. **(B)** The location of crRNAs designed to target sgRNAs of SARS-CoV-2 E, orf7a or N. 5′UTR, the common leader sequence at 5′ flank of sgRNAs of SARS-CoV-2. cr-1, crRNA-1, cr-2, crRNA-2, cr-3, crRNA-3, cr-mix, mixture of crRNA-1,2,3 with equal amount. **(C)** crRNAs screening assay targeting sgRNA of *E*, *orf7a*, or *N*. The fluorescent images at 15 min of the reaction were shown. cr-1, crRNA-1, cr-2, crRNA-2, cr-3, crRNA-3, cr-mix, mixture of crRNA-1,2,3 with equal amount, NC, Negative Control. **(D)** Fluorescent intensities analysis of the fluorescent signal in panel **(C)**. Quantification was performed using ImageJ and analyzed using GraphPad Prism 7.0. **(E)** RT-RPA primers screening assay. The fluorescent images at 15 min of the reaction were shown. **(F–H)** The fluorescent intensity of sg-E **(F)**, sg-orf7a **(G)**, sg-N **(H)** RT-RPA primers screening assay. Quantification was performed using ImageJ and analyzed using GraphPad Prism 7.0. Data were collected from at least three independent experiments and was presented as Mean ± SD, and significance was considered as * *p* < 0.05; **p < 0.01.

After confirming the existence of these sgRNAs, we targeted sgRNAs of *E*, *orf7a* and *N* genes for subsequent investigation due to their functional importance and high abundance in the transcriptome (Kim et al., 2020, 2021). CrRNAs were screened on synthetic DNA fragments using the CRISPR/Cas12a-assisted reporter system. Upon target recognition and cleavage, the non-specific ssDNA cleavage activity will be triggered, and the ssDNA reporter will be cleaved, then the fluorescence signal will be released and detected (Figure 2A). For sgRNA detection, three crRNAs specifically targeting *E*, *orf7a* or *N* (E-cr-1-3, orf7a-cr-1-3, N-cr-1-3) were designed and synthesized separately (Figure 2B). The CRISPR/Cas12a-based detection readout signal relies on the crRNA-guided targeting cleavage efficiency, which is affected by the spacer sequence and secondary structure of crRNA (Kim et al., 2017; Creutzburg et al., 2020). We then assessed the ability of crRNAs to guide Cas12a to detect their targets, individual crRNA and crRNAmix (equal mix work crRNAs targeting each gene, named E-cr-mix, orf7a-cr-mix, N-cr-mix) were included. As the results showed, all tested crRNAs of E, orf7a, or N effectively guided Cas12a to their targets, and crRNAmix generated the strongest fluorescence signal (Figure 2C and Supplementary Figure 2A). The results were further confirmed by the fluorescence signal quantification and a time-course assay (Figure 2D and Supplementary Figure 2B). Thus, crRNAmix targeting each gene (E-cr-mix, orf7a-cr-mix, N-cr-mix) was chosen for subsequent detection.

### Screening of RT-RPA Primers Targeting SARS-CoV-2 Subgenomic RNAs

The RT-RPA was performed to improve CRISPR/Cas12a detection sensitivity. To enable efficient and highly specific amplification of sgRNAs of E, orf7a or N, a total of 27 RT-RPA primers (three forward primers target 5′ untranslated region (5′UTR) and three reverse primers target individual gene body for each target) were designed and synthesized (Supplementary Figure 3A). Screening of RT-RPA primers was performed using RNA templates which were generated through *in vitro* transcription (IVT) of synthetic DNA fragments. As is shown, more than one pair of primers were able to mediate sgRNA amplification of E, orf7a, or N (Figure 2E and Supplementary Figures 3B–D). Specifically, for E sgRNA amplification, F3 and R3 primer pair mediated the strongest signal (Figure 2F); the primer pair of F3 and R1 of orf7a sgRNA enabled most efficient target amplification (Figure 2G); primer pair F2 and R2 performed best for N sgRNA amplification (Figure 2H). Thus, F3 + R3 for E sgRNA, F3 + R1 for orf7a sgRNA, and F2 + R2 for N sgRNA were chosen as the most favorable amplification primer pairs for subsequent experiments.

### Detection Sensitivity of CRISPR-actCoV on SARS-CoV-2 Subgenomic RNAs

**FIGURE 3:**
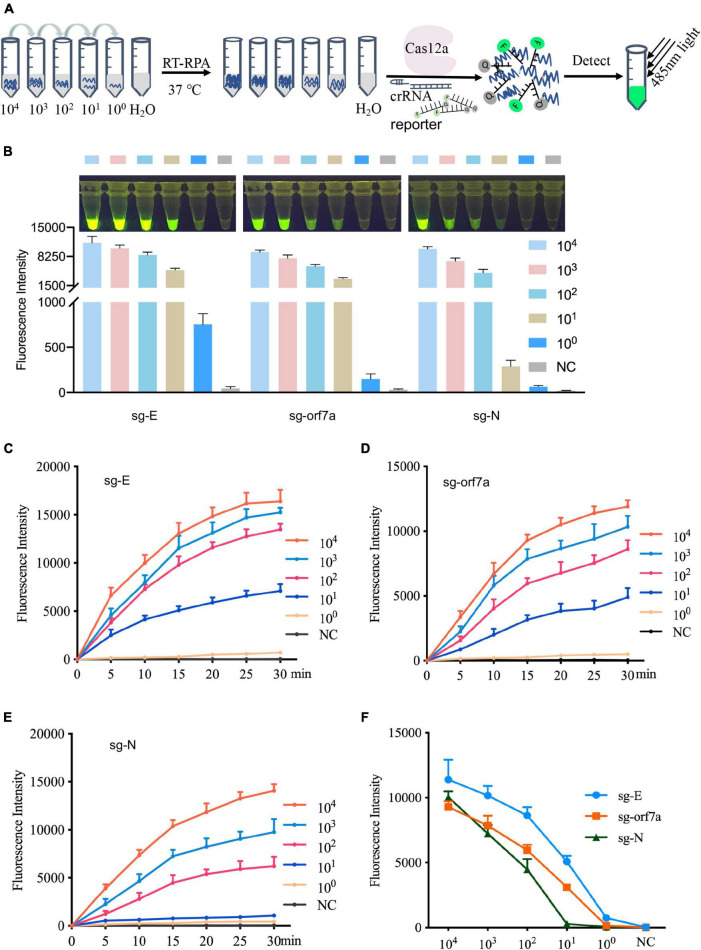
Evaluate the sensitivity of the CRISPR-actCoV detection system. **(A)** An illustration of sensitivity evaluation assay using serially diluted sgRNA. Gradually diluted sgRNAs were subjected to RT-RPA followed by CRISPR/Cas12a mediated fluorescence reporter assay. **(B)** CRISPR-actCoV sensitivity evaluation assay. The fluorescent images (top panel) and intensities (bottom panel) at 15 min of the reaction were shown. Quantification of fluorescent images was performed using ImageJ. **(C–E)** A time-course assay of CRISPR-actCoV sensitivity in detecting sg-E **(C)**, sg-orf7a **(D)**, or sg-N **(E)**. The data was collected in a 5 min interval through 30 min. **(F)** Comparison of detection sensitivity of CRISPR-actCoV targeting sg-E, sg-orf7a, sg-N. Data were collected from at least three independent experiments and was presented as Mean ± SD.

To validate the detection sensitivity of CRISPR-actCoV, RNA fragments containing sgRNA of *E, orf7a*, or *N* genes were prepared by IVT and serially diluted for RT-RPA and subsequent fluorescence detection (Figure 3A). As the results of the fluorescence reporter assay showed, sgRNAs of E and orf7a can be detected clearly at 10 copies; however, for sgRNA of N, the fluorescence signal was not obvious at 10 copies (Figure 3B and Supplementary Figure 4). The results of sgRNAs detection sensitivity were further confirmed by a time-course assay (Figures 3C–E). Among the three detection targets, the fluorescent signal against sgRNA of the *E* gene is the strongest (Figure 3F).

### CRISPR-actCoV Can Detect SARS-CoV-2 Subgenomic RNAs With High Specificity

**FIGURE 4:**
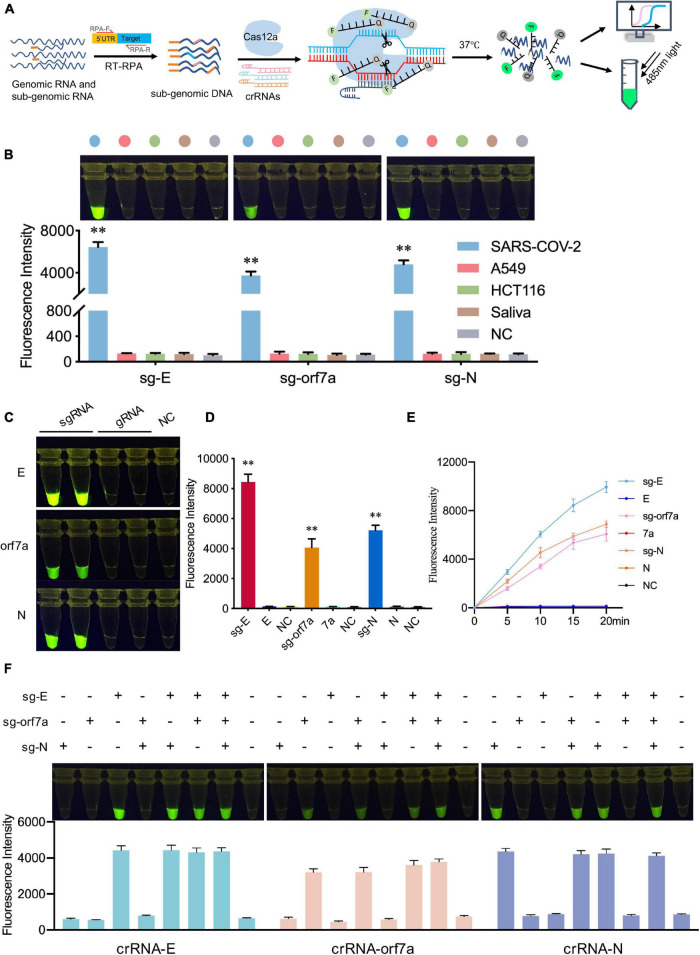
CRISPR-actCoV can detect SARS-CoV-2 sgRNAs with high specificity. **(A)** Schematic illustration of the specificity assessment workflow. SARS-CoV-2 sgRNAs were amplified by RT-RPA using specific primers and subjected to CRISPR/Cas12a reporter system applied with specific crRNAs and ssDNA reporter. **(B)** Specificity validation of CRISPR-actCoV by distinguishing SARS-CoV-2 sgRNAs with background nucleic acid from human cell lines (A549, HCT116) or normal human saliva. The fluorescent images and intensities at 15 min of the reaction were shown. **(C)** Specificity assessment of CRISPR-actCoV by detecting samples containing SARS-CoV-2 sgRNA or gRNA. The fluorescent images at 15 min of the reaction were shown. **(D)** The fluorescent intensities of data in panel **(C)** were quantified by ImageJ and visualized with GraphPad. All the error bars were determined from three independent experiments. **(E)** A time-course assay of SARS-CoV-2 sgRNAs and gRNAs detection. **(F)** CRISPR-actCoV specificity assessment by detecting cross-reaction between different SARS-CoV-2 sgRNAs. sgRNA of E, orf7a, or N were detected individually or simultaneously in a reaction. The fluorescent images and intensities at 15 min of the reaction were shown. Data were collected from at least three independent experiments and was presented as Mean ± SD. The significance was considered as **p < 0.01.

The specificity of CRISPR-actCoV was also assessed. In the CRISPR-actCoV detection system, DNA substrates were specifically generated by RT-RPA using specific primer pairs, targeting specific crRNAs further ensured the specificity of the following CRISPR/Cas12a mediated fluorescence reporter system (Figure 4A). Here, we evaluated the specificity of CRISPR-actCoV in three aspects: the ability to distinguish sgRNA of SARS-CoV-2 from human background nucleic acid; the ability to distinguish subgenomic RNA from genomic RNA; and the ability to enable detection of the specific target across several subgenomic RNAs. Firstly, the ability of CRISPR-actCoV to distinguish the target nucleic acid from background human nucleic acid was validated. The nucleic acid extracted from two cultured human cell lines and normal human saliva was used as background. As is shown, for E, orf7a, and N, only samples containing target sgRNA showed a robust fluorescence signal, whereas no fluorescence signal was detected in background samples (Figure 4B and Supplementary Figure 5A). Subsequently, the specificity of CRISPR-actCoV against sgRNA was confirmed by its failure to detect gRNA. As illustrated by fluorescent images and fluorescent intensities, fluorescent signals were only detected in samples containing target sgRNAs, but not in samples containing gRNAs or NC (Figures 4C,D). A time-course assay further confirmed that CRISPR-actCoV could discriminate target sgRNAs with gRNAs (Figure 4E and Supplementary Figure 5B). Further, to rule out the possibility of detection signal noise between different sgRNAs, the RNA mixtures containing sgRNAs of *E*, *orf7a*, or *N* genes were detected individually or simultaneously. The detection results showed that no cross-activity was detected among these targets (Figure 4F and Supplementary Figure 6). Taken together, these results indicated the high specificity of CRISPR-actCoV in SARS-CoV-2 sgRNAs detection.

### Evaluation of CRISPR-actCoV in Clinical Samples

**FIGURE 5:**
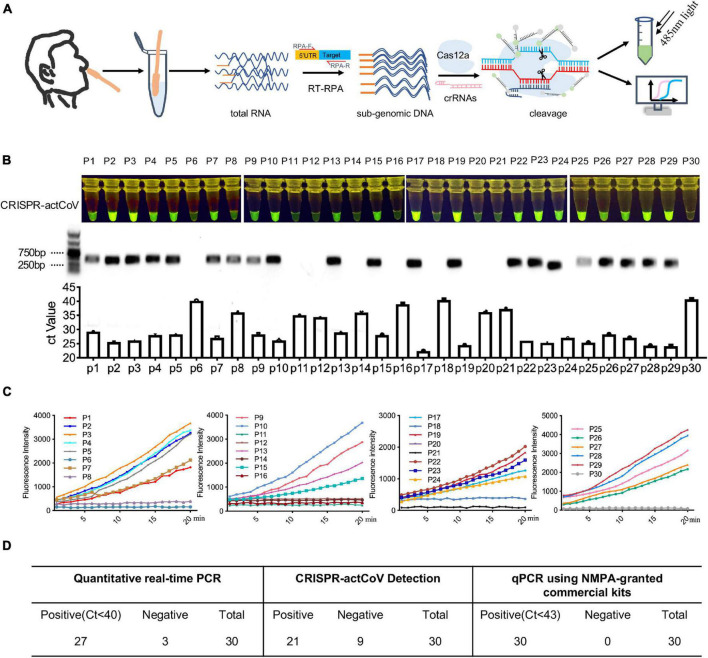
CRISPR-actCoV mediated SARS-CoV-2 sgRNAs detection in clinical samples. **(A)** An illustration of the workflow of CRISPR-actCoV mediated SARS-CoV-2 sgRNAs detection in clinical samples. **(B)** SARS-CoV-2 detection in 30 clinical samples using CRISPR-actCoV (top panel), PCR of sgRNAs (middle panel), and qRT-PCR of gRNA (bottom panel). *E* gene was chosen as the detection target. **(C)** A time-course assay of SARS-CoV-2 sgRNA detection by CRISPR-actCoV in 30 clinical samples. **(D)** The results of CRISPR-actCoV detection in clinical samples compared with real-time PCR.

Finally, we performed CRISPR-actCoV in clinical samples to detect sgRNAs of SARS-CoV-2. The workflow is shown in Figure 5A. All clinical samples were collected and treated according to the WHO and CDC recommended operation for COVID-19. Briefly, nasopharyngeal swab samples were collected from all patients at admission. The clinical sample RNA was extracted using a QIAamp RNA Viral Kit (Qiagen, 52904) in a biosafety level II laboratory. DNA fragments containing SARS-CoV-2 sgRNA were generated by RT-RPA using specific primer pairs. The RT-RPA products were subjected to CRISPR/Cas12a mediated fluorescence reporter assay, and the results can be read by a plate reader or by naked eyes under 485nm light (Figure 5A). Here, we chose the sgRNA of the E gene as the detection target due to its high detection sensitivity. CRISPR-actCoV was performed to detect sgRNA of *E* gene in 30 clinical samples, among all the tested samples, 21 samples were determined to be sgRNA positive, the results were confirmed by DNA electrophoresis and Sanger sequencing (Figure 5B and Supplementary Figure 8). The results of a time-course assay showed that the detection signal could be read in 15 min (Figure 5C and Supplementary Figure 7). QRT-PCR assay was also performed to detect gRNA of SARS-CoV-2 using a research only kit (Vazyme, China) and an NMPA-granted commercial kit (liferiver, China) in parallel. Using research only detection kit, gRNA of *E* gene was targeted, 27 samples had a Ct value below 40, 3 samples had a Ct value above 40, specifically, P6 had a Ct value of 40.2, P18 had a Ct value of 40.5, P30 had a Ct value of 40.9 (Figures 5B,D). Orf1a/b and N were detected using the NMPA-granted commercial kit, the Ct value of all the 30 tested clinical samples were below 43 (Supplementary Figure 9 and Figure 5D).

## Discussion

Rapid and accurate identification of pathogens (bacterias, viruses, parasites, etc.) is essential for disease control. RT-PCR-based strategies have been successfully applied in SARS-CoV-2 genomic RNA (gRNA) detection (Broughton et al., 2020; Joung et al., 2020; Wang et al., 2020a,c; Wolfel et al., 2020; Wu et al., 2020, 2021). Since the gRNA of SARS-CoV-2 might still be detectable for weeks or months after clinical recovery (Binnicker, 2021; Santos Bravo et al., 2021), detection of gRNA could not discriminate between actively replicating virus and remnant viral RNA. In contrast, subgenomic RNAs (sgRNAs) of SARS-CoV-2 are only produced and transcribed following host cell infection, and specific detection of sgRNA could provide evidence of active viral replication (Rodríguez-Grande et al., 2021). Alexandersen et al. (2020) suggested that sgRNAs may not be an accurate indicator of active viral replication as SARS-CoV-2 sgRNAs could be detected for up to 17 days after disease onset. Nonetheless, the detection of sgRNAs can rule out the possibility of RNA contamination and indicate viral replication in tested samples. RT-PCR-based detection of SARS-CoV-2 sgRNAs in animal models, hospitalized patients and patients’ excrement have been reported by many researchers (Rodríguez-Grande et al., 2021; Speranza et al., 2021). Although RT-PCR-based nucleic acid detection methods have been considered as the current gold standard for clinical diagnosis of COVID-19, they are not suitable for simple, rapid, and point of care (POC) molecular diagnostics due to the requirement of experiment equipment and professional technician (Kaminski et al., 2021).

Ideal pathogen detection strategies should be rapid, sensitive, specific, cost-effective, and instrument-free (Wang et al., 2020e). In recent years, CRISPR/Cas systems have been repurposed for next-generation diagnosis applications due to their sensitivity, specificity, flexibility and simplicity. The major Cas proteins involved in nucleic acid detection are Cas9, Cas13, Cas14 and Cas12,the discovery of the collateral nuclease activities of Cas13, Cas14, and Cas12 robust the development of CRISPR/Cas based nucleic acid detection (Gootenberg et al., 2017; Chen et al., 2018; Harrington et al., 2018; Li et al., 2018a; Wang et al., 2020e). Upon specific target recognition by Cas13, Cas14, or Cas12, collateral cleavage of irrelevant single-strand (ssRNA), single-strand DNA (ssDNA), or double-strand DNA (dsDNA) were triggered, respectively. CRISPR/Cas12a based detection systems have been well established for detecting various targets, including bacteria, viruses, cancer mutations, and others (Li et al., 2018b,^2019^; Wang et al., 2020d; Xiong et al., 2020; Lee Yu et al., 2021; Ma et al., 2021). In the current study, we developed a CRISPR-based active SARS-CoV-2 detection strategy (CRISPR-actCoV), enabling rapid, accurate, sensitive and specific detection of SARS-CoV-2 sgRNAs. By targeting sgRNAs of E, orf7a, and N, efficient crRNAs and specific RT-RPA primers were screened, the performance of CRISPR-actCoV was also evaluated. CRISPR-actCoV enables detection of sgRNAs at 10 copies in 35 min with high specificity, and the detection results can be read with naked eyes. This study provides an alternative for rapid and accurate detection of active SARS-CoV-2 and supports that detection of sgRNAs could be considered an indication of active virus replication/active infection.

## Data Availability Statement

The original contributions presented in the study are included in the article/Supplementary Material, further inquiries can be directed to the corresponding author/s.

## Author Contributions

XW, ML, and XH conceived the study. XW, XL, YL, and XH designed the experiments. XL and XW performed the experiments with the assistance of PM, XW, YS, YL, BY, and LW. ML collected and processed the materials. XL, YL, XM, XH, and XW wrote the manuscript. All authors contributed to the article and approved the submitted version.

## Conflict of Interest

The authors declare that the research was conducted in the absence of any commercial or financial relationships that could be construed as a potential conflict of interest.

## Publisher’s Note

All claims expressed in this article are solely those of the authors and do not necessarily represent those of their affiliated organizations, or those of the publisher, the editors and the reviewers. Any product that may be evaluated in this article, or claim that may be made by its manufacturer, is not guaranteed or endorsed by the publisher.
