# Supplementary material for: Rapid and Specific Detection of Active SARS-CoV-2 With CRISPR/Cas12a
Source: Front Microbiol. 2022 Jan 28;12:820698. doi: 10.3389/fmicb.2021.820698 (PMC8832066; doi:10.3389/fmicb.2021.820698)
Supplement: Supplementary file 1 [file Data_Sheet_1.pdf]

Supporting Information

Rapid and specific detection of active SARS-CoV-2 with CRISPR/Cas12a

Supplementary Figures and Tables:

RAPID AND SPECIFIC DETECTION OF ACTIVE SARS-COV-2 WITH CRISPR/CAS12A .. 1

FIGURE S1 2

FIGURE S2 3

FIGURE S3 4

FIGURE S4 5

FIGURE S5 6

FIGURE S6 7

FIGURE S7 8

FIGURE S8 9

TABLE S1 10

TABLE S2 11

TABLE S3 12

TABLE S4 13

TABLE S5 14

Figure S1

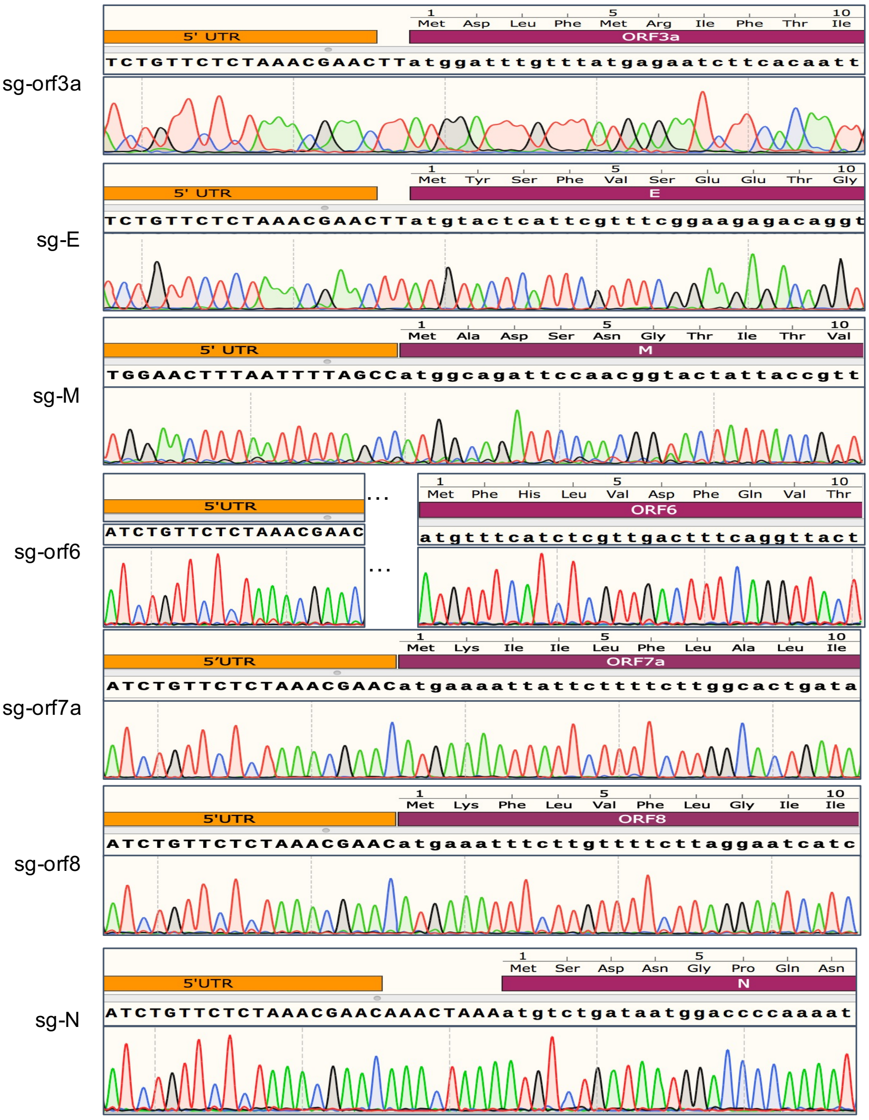

Supplemental figure 1. Chromatograph of Sanger sequencing of amplified SARS-CoV-2 sgRNAs. sg-orf3a, sg-E, sg-M, sg-orf6, sg-orf7a, sg-orf8, sg-N, the subgenomic RNA of *orf3a*, *E*, *M*, *orf6*, *orf7a*, *orf8*, *N* gene.

Figure S2

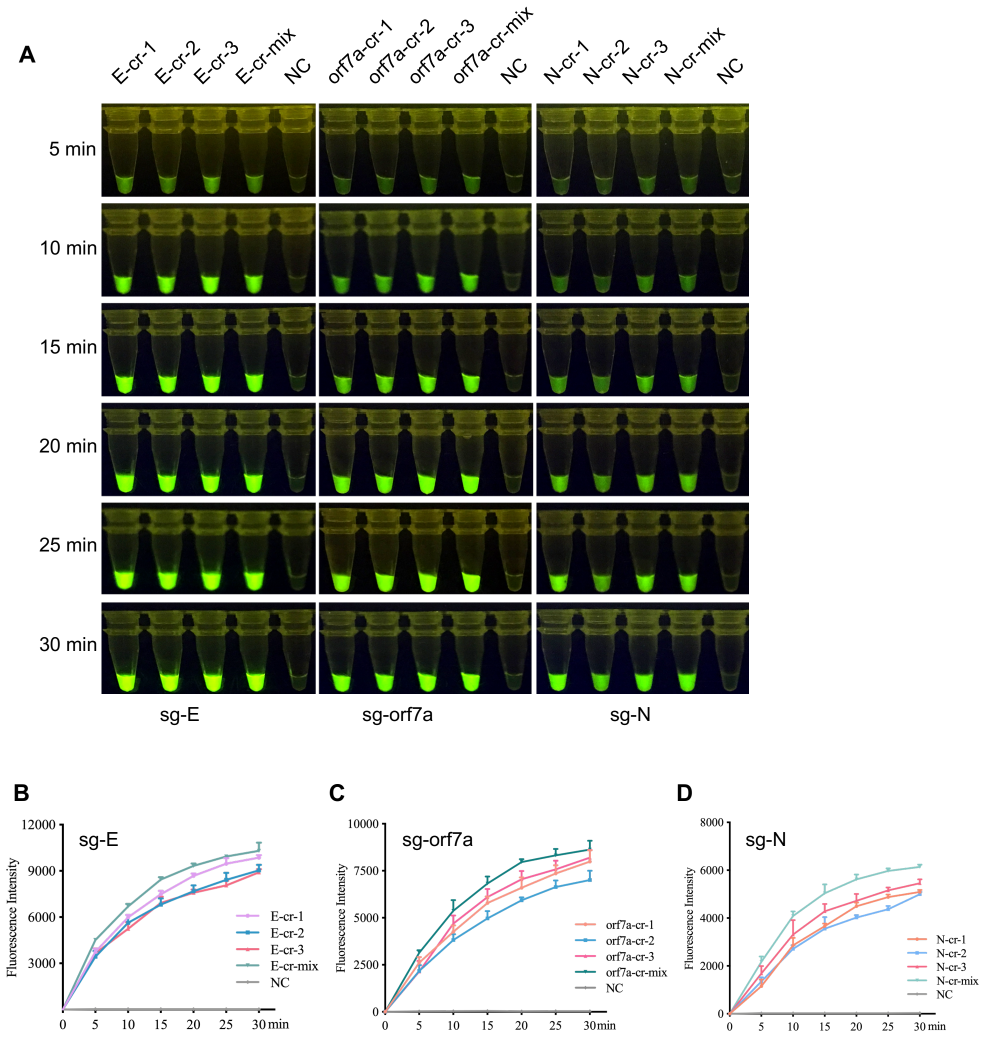

Supplemental figure 2. Screening of crRNAs targeting SARS-CoV-2 *E*, *orf7a*, or *N* gene. (A) Fluorescent image of crRNAs screening in a 30 min time-course. cr-1, crRNA-1, cr-2, crRNA-2, cr-3, crRNA-3, cr-mix, mixture of crRNA-1,2,3, NC, Negative Control. sg-E, sg-orf7a and sg-N, synthetic DNA fragments containing the sgRNA sequence of *E, orf7a* and *N* separately. (**B-D)** The dynamics of the fluorescent signal of crRNAs screening assay targeting sgRNAs of E **(B)**, orf7a **(C)**, N **(D)** over a 30-min time course. Data was presented from at least three individual experiments and error bars indicate mean ± SD.

Figure S3

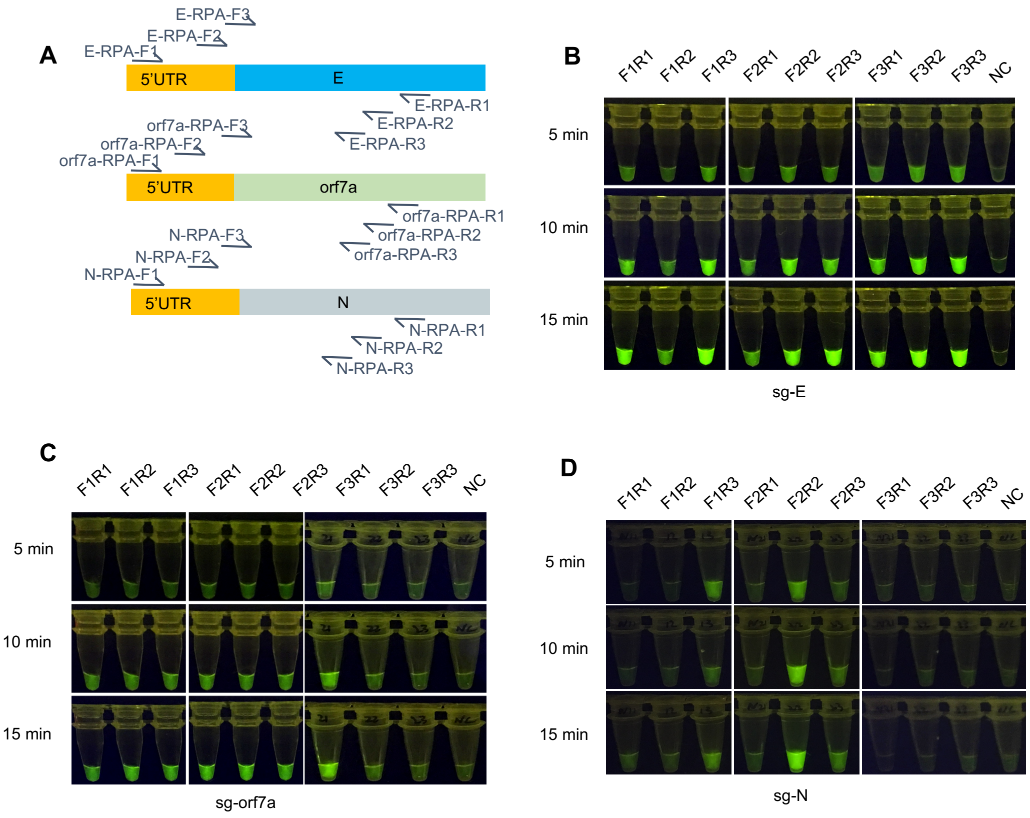

Supplemental figure 3. Fluorescent image of RT-RPA primers screening for efficient and specific SARS-CoV-2 E, orf7a or N sgRNA amplification. (A) Design and location of RT-RPA primers for specific sgRNA amplification. Three forward primers targeting 5'UTR and three reverse primers targeting individual target were designed. **(B-D)** Fluorescent images of RT-RPA primers for E **(B)**, orf7a **(C)**, N **(D)** sgRNA amplification over a 15-min time course.

Figure S4

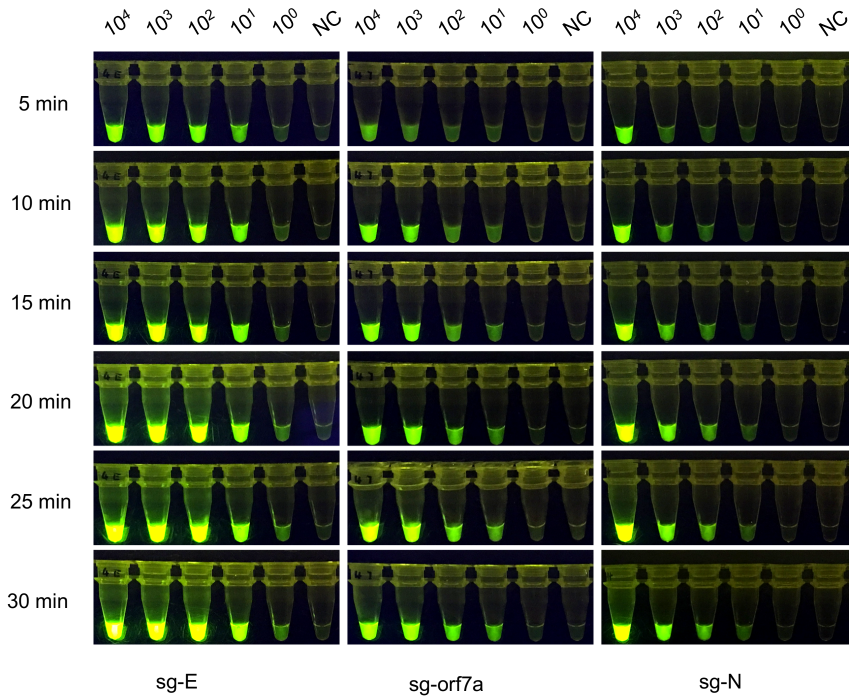

Supplemental figure 4. Fluorescent images of the CRISPR-actCoV detection sensitivity. In vitro transcribed RNA fragments containing sg-E, sg-orf7a, and sg-N was serial diluted and subjected to CRISPR/Cas12a mediated detection. The sensitivity evaluation of CRISPR-actCoV was assayed in a 30-min time course.

Figure S5

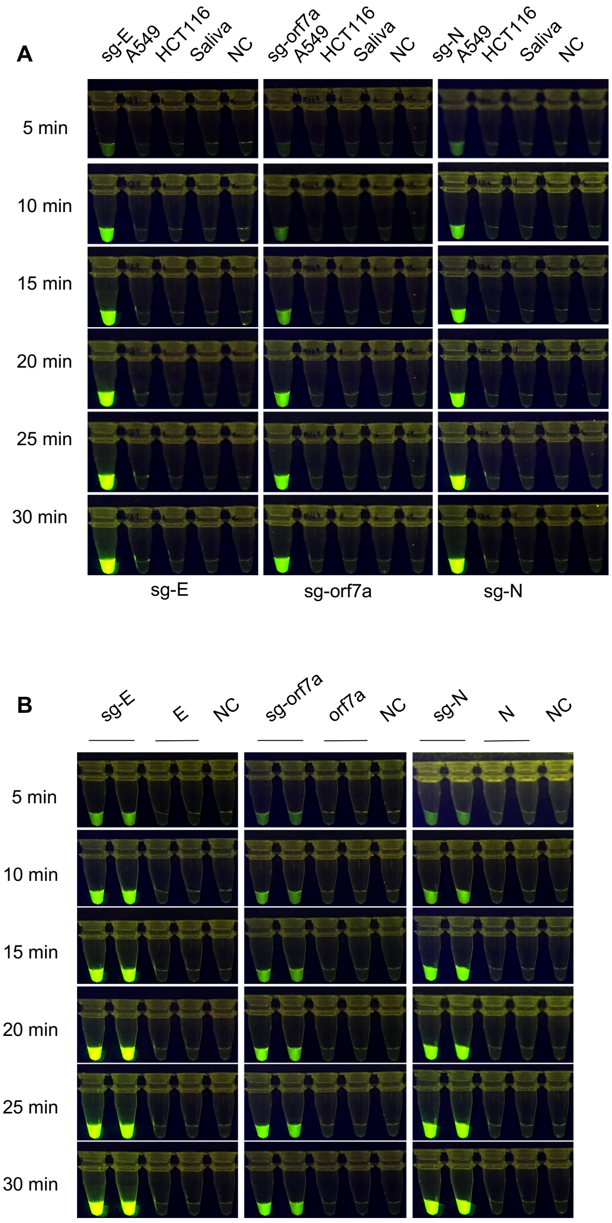

Supplemental figure 5. The fluorescent images of CRISPR-actCoV specificity validation by detecting SARS-CoV-2 sgRNAs from background nucleic acid and genomic RNAs. (A) A time-course assay of CRISPR-actCoV specificity evaluation by detecting SARS-CoV-2 sgRNAs from background nucleic acid. Two human cell lines (A549 and HCT116) and human saliva were used as human background nucleic acid. (B) The fluorescent images of CRISPR-actCoV specificity assessment by detecting SARS-CoV-2 sgRNAs from gRNAs. The detection was performed in a 30-min time course.

Figure S6

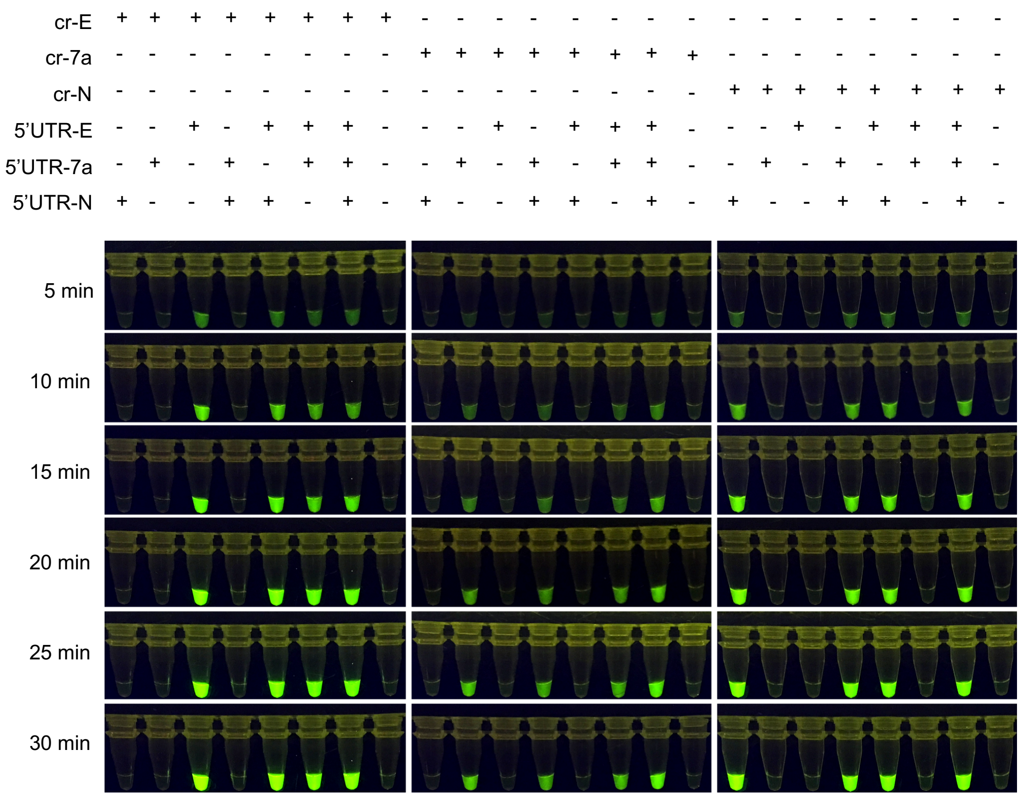

Supplemental figure 6. The specificity evaluation of CRISPR-actCoV by detecting cross-reaction of SARS-CoV-2 sgRNAs. sgRNAs of *E*, *orf7a*, or *N* were detected individually or simultaneously in a reaction. The fluorescent images of the reaction through 30 min with a 5 min interval were shown.

Figure S7

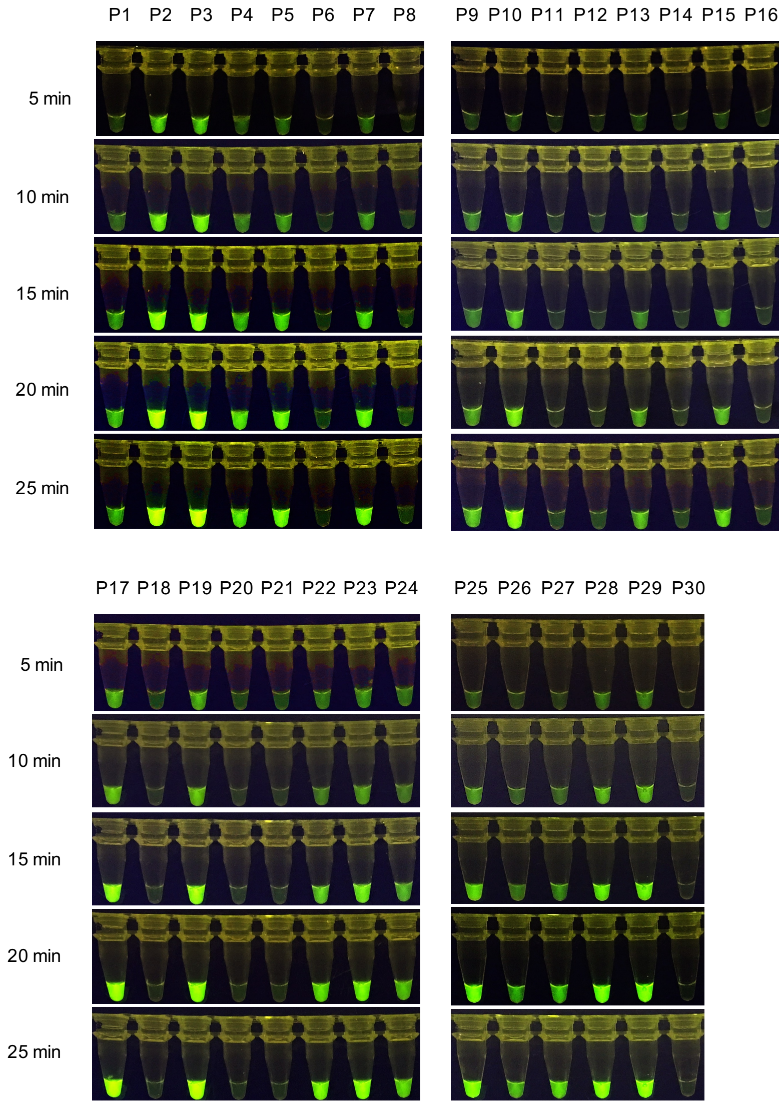

Supplemental figure 7. The fluorescent images of CRISPR-actCoV detection in 30 clinical samples. sgRNA of *E* gene were chosen to be target in clinical sample detection. Fluorescent images were collected in a 5 min time interval through 25 min.

Figure S8

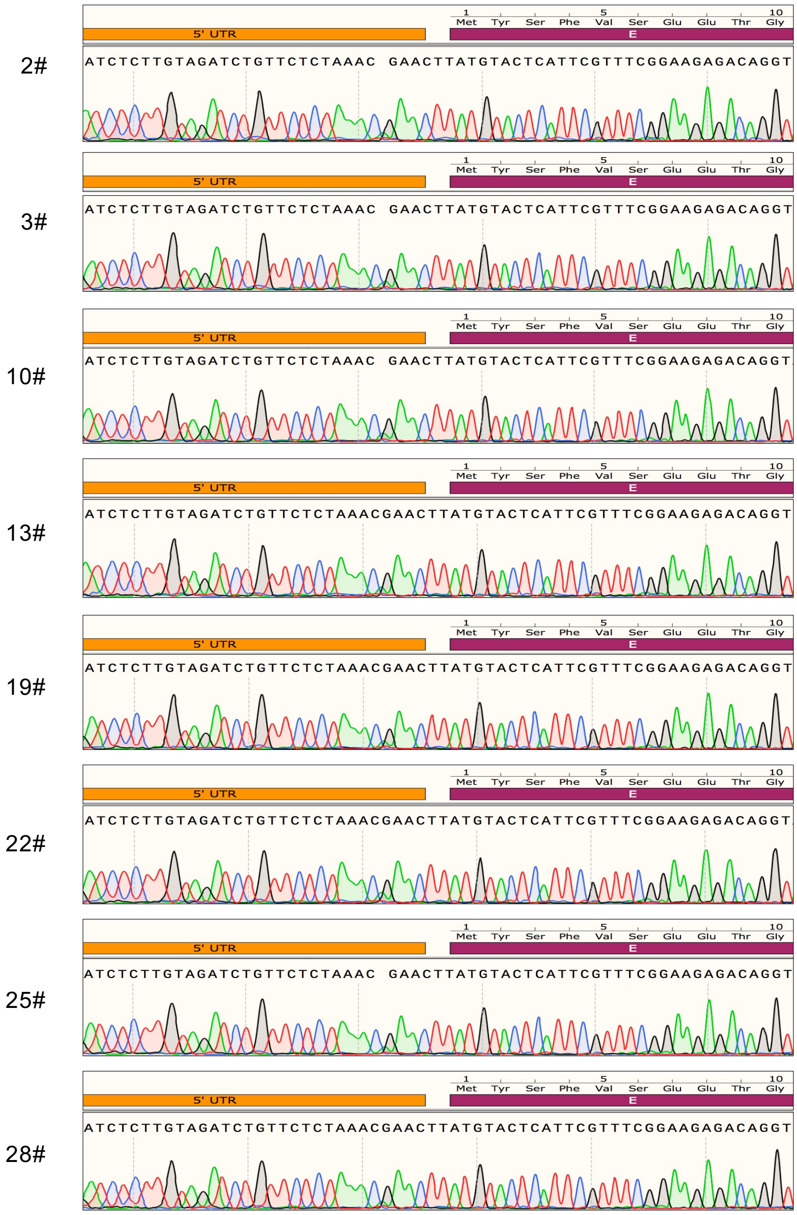

Supplemental figure 8. Several representative chromatographs of Sanger sequencing results of SARS-CoV-2 *E* subgenome in clinical samples. cDNA of clinical samples was used as template to amplify the sgRNA sequence, PCR products were subjected for Sanger sequencing.

Table S1

Supplemental table 1. PCR primers used for amplification of SARS-CoV-2 subgenome in cDNA of a diagnosed clinical sample. 5‘UTR-F, forward primer targeting 5’common leader sequence. orf3a-R, E-R, M-R, orf6-R, orf7a-R, orf8-R, N-R, reverse primers targeting gene body of orf3a, E, M, orf6, orf7a, orf8, N.

PCR Primers	Sequence
5’UTR-F	ATTAAAGGTTTATACCTTCCCAGG
Orf3a-R	CAAAGGCACGCTAGTAGTCGTC
E-R	GACCAGAAGATCAGGAACTCTAG
M-R	GCAGTGACAATATTGCTTTGCTTGTACAG
Orf6-R	ATCAATCTCCATTGGTTGCTCTTC
Orf7a-R	GCTTCACACTCAAAAGAAAGACAGAA
Orf8-R	GATGAAATCTAAAACAACACGAACG
N-R	GGCCTGAGTTGAGTCAGCACTG

Table S2

Supplemental table 2. crRNA sequence used for crRNA screening targeting *E*, *orf7a* or *N* gene. E-cr-1–E-cr-3, crRNA1–crRNA3 targeting E. 7a-cr-1–7a-cr-3, crRNA1–crRNA3 targeting orf7a, N-cr-1–N-cr-3, crRNA1–crRNA3 targeting N.

crRNAs	Sequence
E-cr-1	GGAAGAGACAGGTACGTTAATAG
E-cr-2	TTGCTTTCGTGGTATTCTTGCTA
E-cr-3	GTGGTATTCTTGCTAGTTACACT
7a-cr-1	TTGGCACTGATAACACTCGCTAC
7a-cr-2	TCACTACCAAGAGTGTGTTAGAG
7a-cr-3	AAAGAACCTTGCTCTTCTGGAAC
N-cr-1	GTGGACCCTCAGATTCAACTGGC
N-cr-2	ATCGCGCCCCACTGCGTTCTCCA
N-cr-3	CCCAATAATACTGCGTCTTGGTT

Table S3

Supplemental table 3. In vitro transcription primers used for amplification of fragments containing subgenomic RNA sequence of SARS-CoV-2 *E, orf7a* or *N*.

Primers	Sequence
5'UTR-T7-F	GATATCGGATCCCTAATACGACTCACTATAGGAGAATTAAAGGTTTATACCTTCCCAGG
5’UTR-E-T7-R	CGGGCCCTTTAAAAAAAGACCAGAAGATCAGGAACTCTAG
5’UTR-7a-T7-R	CGGGCCCTTTAAAAAAATAGACGTGTTTTACGCCGTC
5’UTR-N-T7-R	CGGGCCCTTTAAAAAAAGAAGTTGTAGCACGATTGCAGC

Table S4

Supplemental table 4. RT-RPA primers used for amplification of sgRNAs of *E*, *orf7a*, *N*. Three forward primers targeting 5'UTR and three reverse primers targeting individual target were designed.

Primers	Sequence
5'UTR-7a RPA-F1	TTTATACCTTCCCAGGTAACAAACCAACCAAC
5'UTR-7a RPA-F2	CCCAGGTAACAAACCAACCAACTTTCGATCTCTTG
5'UTR-7a RPA-F3	TTTCTTGGCACTGATAACACTCGCTACTTGTGAGC
5'UTR-7a RPA-R1	TGATAGACGTGTTTTACGCCGTCAGGACAAGC
5'UTR-7a RPA-R2	TTTACGCCGTCAGGACAAGCAAAAGCAAATTGAG
5'UTR-7a RPA-R3	TTATCAGCTAGAGGATGAAATGGTGAATTGCCC
5'UTR-N RPA-F1	CCAACCAACTTTCGATCTCTTGTAGATCTGTTCTC
5'UTR-N RPA-F2	TTCTCTAAACGAACTTATGTCTGATAATGGACCCC
5'UTR-N RPA-F3	CCCCAAAATCAGCGAAATGCACCCCGCATTACG
5'UTR-N RPA-R1	TCATCTGGACTGCTATTGGTGTTAATTGGAACGCC
5'UTR-N RPA-R2	ATTTAAGGTCTTCCTTGCCATGTTGAGTGAGAGC
5'UTR-N RPA-R3	CATGTTGAGTGAGAGCGGTGAACCAAGACGCAG
5'UTR-E RPA-F1	TTTATACCTTCCCAGGTAACAAACCAACCAAC
5'UTR-E RPA-F2	CCTTCCCAGGTAACAAACCAACCAACTTTCGATC
5'UTR-E RPA-F3	TCCCAGGTAACAAACCAACCAACTTTCGATCTC
5'UTR-E RPA-R1	TATTGCAGCAGTACGCACACAATCGAAGCGCAG
5'UTR-E RPA-R2	CGCACACAATCGAAGCGCAGTAAGGATGGCTAG
5'UTR-E RPA-R3	CAGTAAGGATGGCTAGTGTAACTAGCAAGAATACC

Table S5

Supplemental table 5. Sequences of primers and probe used for qRT-PCR of clinical samples.

primers	Sequence
E_Sarbeco_F1	ACAGGTACGTTAATAGTTAATAGCGT
E_Sarbeco_R2	ATATTGCAGCAGTACGCACACA
E_Sarbeco_P1	ACACTAGCCATCCTTACTGCGCTTCG
